# Sharper displays via field control

**DOI:** 10.1038/s41377-026-02329-y

**Published:** 2026-06-15

**Authors:** Yuguang Ma

**Affiliations:** State Key Laboratory of Luminescent Materials and Devices, South China University of Technology, Guangzhou, 510640 China

**Keywords:** Inorganic LEDs, Optical techniques

*Nature* 652, 349–358 (2026)

https://www.nature.com/articles/s41586-026-10333-w

Pushing quantum dot LEDs (QLEDs) to extreme resolutions—beyond 10,000 pixels per inch—is essential for the next generation of AR/VR headsets. However, as pixels shrink to sub-micrometer sizes, their efficiency typically plummets, a problem long thought to be a simple manufacturing limit. Reporting in *Nature*, Lihua Lin, Fushan Li, and their team reveal that the real culprit is “edge leakage” caused by distorted internal electric fields. To fix this, they developed a high-precision nanoimprinting technique that patterns pixels with unprecedented density (up to 25,400 PPI). More importantly, they introduced specialized nanoparticles to balance the internal electric field, ensuring light is emitted uniformly across the tiny pixel. The results are record-breaking: red QLEDs achieving 26.1% efficiency and exceptional stability at ultra-high resolutions. By treating pixel design as a balance of both shape and electricity, this work provides a blueprint for ultra-sharp, energy-efficient micro-displays that could redefine our digital visual experience.

